# Draft Genome Sequence of *Desulfovibrio* BerOc1, a Mercury-Methylating Strain

**DOI:** 10.1128/genomeA.01483-16

**Published:** 2017-01-19

**Authors:** Marisol Goñi Urriza, Claire Gassie, Oliver Bouchez, Christophe Klopp, Rémy Guyoneaud

**Affiliations:** a Equipe Environnement et Microbiologie, IPREM UMR CNRS 5254, Université de Pau et des Pays de l’Adour, IBEAS, Pau, France; b GeT-PlaGe, Genotoul, INRA Auzeville, Castanet-Tolosan, France; c Plateforme Bioinformatique Genotoul, UR875, Biométrie et Intelligence Artificielle, INRA, Castanet-Tolosan, France

## Abstract

*Desulfovibrio* BerOc1 is a sulfate-reducing bacterium isolated from the Berre lagoon (French Mediterranean coast). BerOc1 is able to methylate and demethylate mercury. The genome size is 4,081,579 bp assembled into five contigs. We identified the *hgcA* and *hgcB* genes involved in mercury methylation, but not those responsible for mercury demethylation.

## GENOME ANNOUNCEMENT

*Desulfovibrio* BerOc1 is a vibrio-shaped motile sulfate-reducing bacterium isolated from the highly oil-contaminated sediments of the Berre lagoon. It has been isolated from an anaerobic enrichment with octadecane as the sole carbon source (1). BerOc1 grows under sulfate reduction, fumarate respiration, and pyruvate fermentation. Lactate, pyruvate, and ethanol are used as carbon sources, but not acetate. BerOc1 is able to methylate and demethylate mercury (2). The strain BerOc1 has already been used as a model organism to better understand the mercury species distribution (3), their isotopic fractionation (4), and the physiology of the methylation (5). The potential of mercury methylation of BerOc1 varied depending on growing conditions (5), with a maximum potential occurring under fumarate respiration, probably because the absence of sulfide allows the inorganic mercury to be more available (6).

The total DNA of BerOc1 was extracted using an UltraClean microbial DNA isolation kit (Mo Bio). The genome was sequenced with 454 Roche Technology, using 454 GS FLEX Titanium version. Sequences were assembled using Newbler 2.6 (454 Life Sciences). We obtained 36 contigs, with a mean depth of coverage of 45×. Contigs were reordered using Promer (7) and the *Desulfovibrio desulfuricans* ND132 genome (RefSeq GenBank accession no. NC_016803) as the template. They were further extended by PCR. The draft genome includes five contigs, with the longest and shortest sequences being 2,803,249 and 2,705 bp, respectively. The total size of the genome is 4,081,579 bp, with a G+C content of 63.79%. The final assembly was annotated using Prokka version 1.10 (8) and identified 3,686 coding regions, three rRNAs, 59 tRNAs, and one transfer-messenger RNA (tmRNA). While no plasmid amplicon was detected, five putative genomic islands (29,653 bp, 27,947 bp, 11,484 bp, 19,691 bp, and 7,691 bp) were predicted using IslandViewer (9). There were three transposases annotated and four integrases; the presence of a bacteriophage is suspected by the presence of prophage-derived endonuclease YokF precursor and some proteins involved in tail constitution. No clustered regularly interspaced short palindromic repeat (CRISPR) could be detected using the CRISPR Recognition Tool (CRT) version 1.0 software (10).

The products of the *hgcA* and *hgcB* genes have been described to be involved in mercury methylation (11). The HgcA protein, belonging to Pterin-binding superfamily, shared 69% identity with HgcA of *D. desulfuricans* ND132 and* Desulfovibrio aespoeensis* (GenBank accession no. NC_014844). Notably, the motif NVWCAAGKG, known to be necessary for mercury methylation, was identical (12). HgcB shared 77% and 63% identity with HgcB of *D. desulfuricans* ND132 and* Desulfovibrio aespoeensis*, respectively. The operon *mer*, involved in methylmercury demethylation, could not be detected in the BerOc1 genome, suggesting that this process is performed through another unknown metabolic pathway.

Mostly all of the *Desulfovibrio* strains tested are able to demethylate mercury, but few strains are able to methylate it (13). The comparison of the genomes of *Desulfovibrio* strains sharing the capacity to produce this highly toxic mercury species with those unable to drive this process will give new tracks in the understanding of methylmercury production by bacteria.

### Accession number(s).

This whole-genome shotgun project has been deposited at DDBJ/EMBL/GenBank under the accession no. LKAQ00000000. The version described in this paper is version LKAQ01000000.
